# Thai nursing students’ experiences of stress, role positioning, and facilitation tone in simulation-based learning: implications for culturally responsive facilitation and debriefing

**DOI:** 10.1186/s41077-026-00472-5

**Published:** 2026-07-18

**Authors:** Trakulwong Luecha, Charunyakorn Viriya, Nattaya Sangsai, Patcharin Poonthawe, Witchaporn Kidsamrong, Warisa Kanbuala, Puntaree Pikulnee, Paweena Tivasiripong

**Affiliations:** 1 Department of Community Nursing, Faculty of Nursing, Burapha University, 169 Long-Hard Bangsaen Road, Tambon Saensuk, Amphur Muang, Chonburi, 20131 Thailand; 2 Department of Paediatric Nursing, Faculty of Nursing, Burapha University, 169 Long-Hard Bangsaen Road, Tambon Saensuk, Amphur Muang, Chonburi, Thailand; 3 Department of Adult Nursing, Faculty of Nursing, Burapha University, 169 Long-Hard Bangsaen Road, Tambon Saensuk, Amphur Muang, Chonburi, Thailand; 4 Department of Maternity Nursing and Midwifery, Faculty of Nursing, Burapha University, 169 Long-Hard Bangsaen Road, Tambon Saensuk, Amphur Muang, Chonburi, Thailand

**Keywords:** Simulation-based learning, Nursing students, Debriefing, Cultural safety, Kreng-jai, Thailand, Reflexive thematic analysis

## Abstract

**Background:**

Simulation facilitation models have developed predominantly in Western educational settings. The ways in which cultural norms, particularly *kreng-jai* and deference to authority, shape nursing student participation in high power-distance contexts have received limited empirical attention.

**Objectives:**

This study examined how stress, role positioning, and facilitator feedback timing and tone interact to shape participation and culturally safe learning in simulation-based nursing education in Thailand.

**Methods:**

Semi-structured interviews were conducted in Thai with 24 third- and fourth-year nursing students in Eastern Thailand between August and October 2024. Interviews lasted 45–60 min and were analysed using reflexive thematic analysis.

**Results:**

Four themes were developed. Entering a New Space positioned simulation as a threshold where visibility intersected with kreng-jai-patterned restraint; students’ reluctance to speak was culturally rational rather than disengagement. Stress as a Double-Edged Sword in Simulation showed that pressure could sharpen recall and prioritisation but could also narrow participation when abrupt feedback arrived mid-scenario. Learning Across Participation, Observation, and Debriefing identified distinct but complementary learning routes: active scenario participants acted under time pressure, whereas observers developed an analytic perspective, with both perspectives converging during debriefing. Facilitation and Cultural Safety found that calm guidance kept kreng-jai compatible with engagement, whereas abrupt correction rendered silence a response to relational risk.

**Conclusion:**

Participation and psychological safety in simulation were not inherent features of instructional design but were interactionally and culturally mediated through facilitation, role positioning, and hierarchical norms.

**Supplementary Information:**

The online version contains supplementary material available at https://doi.org/10.1186/s41077-026-00472-5.

## Introduction

Most simulation facilitation and debriefing frameworks were built in educational settings where students are expected to speak up, ask questions, and engage openly with instructors. That expectation is embedded in how these frameworks are designed. In contexts where hierarchy shapes communication differently, applying these frameworks without adjustment may create conditions in which silence looks like passivity, and passivity is met with responses that make speaking even less likely [12]. This paper examines that problem in the Thai undergraduate nursing context, where culturally patterned silence during simulation has been documented but remains underexamined in the facilitation literature.

The theory–practice gap in nursing education is well recognised. In Thailand, second-year students have described early clinical placements as more demanding than expected: knowing the steps of care in class did not prepare them for carrying those steps out in front of a real patient [3, 31]. SBL has been widely adopted to help close that gap, giving students a structured space to work through clinical situations before the stakes become real [1, 10]. For some students, it works: simulation becomes the point at which the nursing role shifts from something studied to something practised [26, 33]. Whether it reaches that point depends less on the scenario itself than on how the session is run — the facilitator’s conduct, the timing of feedback, and what students judge they can safely do and say [12, 22].

In Thailand, those conditions are also shaped by kreng-jai — the tendency to avoid causing discomfort or embarrassment to those in authority [2, 11]. In educational settings characterised by high power distance [15]— where the social distance between instructor and student is both real and felt — kreng-jai shapes participation in ways that standard debriefing prompts were not designed to navigate. Students who would speak freely in a lower power-distance setting may calculate, in this one, that the relational cost of speaking outweighs the learning benefit. Students who remain quiet during debriefing are not necessarily disengaged. Silence may be the more cautious option — a way of avoiding exposure of a peer’s error, or of one’s own, in a room where such things carry real interpersonal cost [16, 30]. Silence, in this sense, can be meaningful rather than empty. A facilitator who reads it only as passivity may respond in ways that entrench it rather than address it.

This matters across the whole session. Pre-briefing, scenario, and debrief are usually presented as separate stages, but students do not always experience them that way — the tone established early tends to carry [13, 25]. Psychological safety can erode mid-session: a sharp correction, a debrief that feels more like an audit than a conversation, a facilitator whose manner signals that mistakes are problems rather than material for learning [12, 14]. In contexts where hierarchy and face concerns are already present, these moments may carry more weight than the same moments would in other settings.

Published SBL studies in Thailand have focused mainly on satisfaction and confidence as outcomes [18, 33]. These matter, but they say little about what students are navigating during the session itself — whether to speak, how role allocation shapes engagement, or how facilitation conduct mediates participation where cultural and relational considerations are always in the background. This study drew on in-depth interviews with Thai undergraduate nursing students to examine those dimensions of the simulation experience. The findings are intended to inform facilitation and debriefing practice in settings where cultural context shapes participation — and to contribute to a growing body of work questioning whether simulation pedagogy developed in one educational tradition can be adopted, unchanged, in another.

## Research question

How did Thai undergraduate nursing students experience simulation-based learning, and in what ways did stress, role positioning, and facilitator interactions shape their participation and psychological safety across the simulation flow?

## Methodology

### Research design

This paper reports an interpretive qualitative study of Thai undergraduate nursing students’ experiences of simulation-based learning. The study was informed by an experiential orientation and focused on how students made sense of stress, role positioning, and facilitation within the simulation environment. Reflexive thematic analysis [6] was used as the analytic framework. In this study, themes were treated as interpretive patterns of shared meaning developed through close engagement with the data rather than as summaries of surface content.

The study adopted an interpretivist orientation, treating participants’ accounts not as transparent reports of events but as meaning making shaped by cultural and institutional context. Although the analysis remained grounded in students’ descriptions of their simulation experiences, it was interpretive rather than merely descriptive. Researcher subjectivity was therefore not bracketed out but treated as analytically relevant and examined throughout the analytic process [7].

The interviews were conducted as part of a broader programme of research at the same institution. This paper reports the qualitative findings as a standalone interpretive analysis, with no integration of quantitative data.

### Setting and SBL implementation

The study was conducted in an undergraduate nursing programme in Eastern Thailand, with SBL embedded across clinical practicum courses covering adult, paediatric, maternal and child, and community nursing. Sessions were delivered to large cohorts using a rotation-based format across multiple rooms. One instructor typically oversaw two to three concurrent student groups during large-cohort simulation days, moving between groups to lead debriefing at the close of each scenario run. Within each scenario, four to six students acted as scenario participants while the remainder took observer roles. Roles were rotated across repeated runs to support equitable exposure. Sessions followed a consistent structure: pre-brief (5–10 min), scenario run (10–15 min) and debrief (20–30 min). Debriefing used the GAS model (Gather–Analyse–Summarise). Facilitators had completed institutional training in simulation-based teaching and had experience in both skills laboratory and ward settings.

### Participants and recruitment

Purposive sampling with a maximum-variation approach was used to recruit 24 third- and fourth-year undergraduate nursing students who had recently participated in SBL. Variation was sought across practicum areas, most recent simulation role (scenario participant or observer), and extent of prior SBL exposure. Recruitment materials were distributed via campus noticeboards, QR-code posters, and the course learning management system. Expressions of interest were directed to the research team rather than teaching staff. Course instructors had no involvement in recruitment, interviewing, or student assessment for this study. Participation was voluntary and uncompensated.

The final sample size was judged adequate using the principle of information power rather than a predetermined numerical target [20]. The study had a focused aim, a relatively specific participant group, and rich interview data generated through 45–60-minute semi-structured interviews. Recruitment was stopped at 24 interviews because subsequent interviews mainly deepened, rather than redirected, the developing analysis. This judgement was made during coding, memo writing, and team discussions about the emerging themes. Consistent with reflexive thematic analysis, sample adequacy was considered in relation to the richness, relevance, and analytic contribution of the interviews, rather than statistical representativeness [7]. Including both third- and fourth-year students enabled the analysis to reflect variation in clinical exposure and prior simulation experience within the same institutional SBL programme.

### Instruments

A semi-structured interview guide was developed with reference to the simulation-based learning (SBL) and experiential learning literature and organised around the typical phases of simulation: pre-briefing, scenario participation or observation, and debriefing. The guide explored students’ sense of preparedness, cognitive and emotional load, role allocation and the observer experience, perceptions of debriefing quality, authenticity and fidelity, transfer to clinical learning, and views on assessment-related implications.

The guide was reviewed by three experts in simulation pedagogy and nursing education, who assessed clarity, relevance, and coverage. Their feedback was used to refine the questions. The guide was then piloted with two students who were not included in the final analysis, resulting in minor wording adjustments [24]. No incentives were offered in order to minimise undue influence on participation.

The interview guide moved from brief contextual questions to the simulation experience itself. It began with background questions, including age, year of study, and current study or life context, before exploring prior exposure to SBL and perceptions of the classroom learning climate. Participants were then asked about implementation details, including their role in the simulation (active scenario participant or observer), the number and length of scenarios, the adequacy of pre-briefing, and available resources or fidelity. They were then invited to reflect on learning, including clinical judgement and confidence, and to identify areas for improvement. A brief satisfaction check was used only to open conversation and prompt richer accounts; it was not treated as quantitative data.

### Procedure

Students who had completed the institutional SBL survey were invited, at the end of that questionnaire, to volunteer for a follow-up interview. Those who opted in accessed study information via a QR code and provided electronic consent before scheduling. Interviews were conducted in Thai, one-to-one, either face-to-face in a private campus room or remotely, according to participant preference, between four and eight weeks after students’ most recent simulation session. Four members of the research team (TL, PP, CV, NS) conducted interviews using the same guide. Interviews lasted 45–60 min and were audio-recorded with permission and transcribed verbatim. Quotations in this paper were translated into English by a bilingual researcher and reviewed by a second bilingual co-author for meaning equivalence [27, 29, 32]. Brief field notes on setting and participants’ affect informed interpretation during analysis.

### Data analysis

Reflexive thematic analysis was used to develop patterns of meaning across the interview dataset [6, 7]. This approach was appropriate because the study sought to interpret how students made sense of simulation, participation, and facilitation, rather than to quantify code frequency or produce a consensus-coded description. Themes were treated as analytic constructions developed through sustained engagement with the data, the research question, and the study context. Researcher subjectivity was therefore not treated as a source of error to be removed, but as part of the interpretive work to be examined reflexively.

Transcripts were managed in QDA Miner. Analysis began with repeated reading to develop familiarity with the range and texture of participants’ accounts. TL and PP generated initial codes as an interpretive and iterative process, attending to how students described stress, role positioning, observation, participation, and facilitator feedback. Codes were revisited, merged, and reworked as later interviews complicated earlier readings. Regular analytic meetings served as critical dialogue, allowing the team to surface assumptions, test emerging interpretations, and return to original extracts to examine coherence and distinctiveness. Key analytic decisions were documented in dated memos throughout. In keeping with reflexive thematic analysis, we did not calculate inter-coder reliability. Analytic quality was supported through team discussion, repeated return to the transcripts, and memoing of key analytic decisions [8].

### Researcher reflexivity

The research team comprised nurse educators with experience designing and facilitating SBL. This positioning provided contextual sensitivity to the simulation environment and the interactional cues students described, while also carrying the risk of normalising familiar facilitation practices or reading students’ accounts through the lens of what simulation is supposed to do. TL and PP kept reflexive memos throughout coding and theme development to track assumptions, moments of interpretive friction, and points where early readings did not hold up against later interviews. These were used in analytic dialogue to examine how the team’s professional roles and cultural insider status shaped interpretation — particularly around silence, deference, and kreng-jai, where over-reading was a recognised risk. A senior qualitative researcher not involved in students’ teaching reviewed emerging theme summaries and pressed the team to maintain boundaries, resist over-reading, and hold open alternative interpretations where the data were ambiguous.

### Ethical considerations

Ethical approval was obtained from the Burapha University Institutional Review Board (HS046/2567[E3]). Participation was voluntary, and electronic informed consent was obtained before interviews were scheduled. To reduce perceived coercion, recruitment and interviewing were separated from teaching and grading responsibilities, and interviews were conducted after formal course assessments had been completed. Data were de-identified and stored securely.

### Analytic quality

Analytic quality was addressed through practices aligned with reflexive thematic analysis and the study’s interpretivist orientation, rather than by applying Lincoln and Guba’s trustworthiness criteria as a checklist. Quality was supported through verbatim transcription, repeated return to original extracts, dated analytic memos, and regular team discussions about developing interpretations. The team paid particular attention to theme coherence and boundaries, especially where accounts of silence, hierarchy, feedback, and relational risk could be interpreted in more than one way. In these cases, we returned to participants’ words and discussed whether the interpretation remained grounded in the data or reflected researcher assumptions. Contextual detail about the setting, participants, and SBL implementation is provided to help readers judge the relevance of the findings to other contexts [8].

## Results

Twenty-four students took part. Most were female, aged between 20 and 24 years, and included both third- and fourth-year students. This mix allowed variation in prior simulation exposure while still reflecting the make-up of the wider cohort. Analysis focused on how students described entering, participating in, and making sense of simulation across the pre-brief, scenario, and debriefing flow, with particular attention to role positioning (active scenario participant or observer) and facilitation as experienced within Thai classroom culture.

### Learners’ experiential overview of SBL

Students rarely described simulation as just another class. Instead, they spoke about SBL as a distinctive learning space with a ward-like feel and a heightened sense of visibility, shaped by equipment, time constraints, and being watched by peers and instructors. As one participant noted, *“It was like caring for a real patient through role-play”* (Case 8). When describing participation—particularly in debriefing—students often framed silence and reluctance to correct peers as socially and culturally appropriate rather than as disengagement: *“When the teacher had already spoken*,* I didn’t add my view—I didn’t want to look impolite”* (Case 7). Overall, students remembered simulation through its emotional and relational weight as much as its technical content, providing context for the themes presented below.

### Thematic analysis

Reflexive thematic analysis developed four themes: (1) Entering a New Space, (2) Stress as a Double-Edged Sword in Simulation, (3) Learning Across Participation, Observation, and Debriefing, and (4) Facilitation and Cultural Safety (Table 1). Entering a New Space positioned simulation as a “third space” between classroom and ward, shaped by realism, pressure, and culturally patterned silence. In Stress and Roles as Catalysts for Learning, students described nervousness as having two effects. In some situations, it sharpened focus and helped them recall procedural steps more quickly. In others, it reduced their willingness to speak. Learning was also shaped by role positioning. Those in active scenario roles described pressure, responsibility, and the immediacy of decision making, whereas observers described more cognitive space to notice patterns, compare actions, and think ahead.

**Table 1:** Themes, sub-themes, illustrative meaning, and example quotations

Theme	Sub-theme	Illustrative Meaning	Example Quotations (EN, Case No.)
Theme 1: Entering a New Space—Navigating from Classroom to Simulation	Sub-theme 1.1: The weight of pressure	Students described early simulation encounters as emotionally weighty, with stress heightened on entering the room and intensifying when taking an active scenario participant role rather than observing.	“Every subject felt stressful… when I entered the simulation, the pressure was already there” (Case 7) “When I was the one inside the scenario, it was more stressful than when I was just observing” (Case 7)
	Sub-theme 1.2: Realism as rehearsal for practice	Students valued simulation as rehearsal for practice, allowing them to attempt patient care independently, make mistakes safely, and recalibrate before clinical placements.	“It let me practice caring for patients on my own; it felt like I could really try and then see how to improve” (Case 8) “When I went in, it was as if I was already caring for a patient, not just studying in class” (Case 12)
	Sub-theme 1.3: Silence as a cultural stance	Hesitation to speak—particularly to correct peers—was framed as respectful restraint shaped by kreng-jai, protecting face and maintaining harmony rather than disengagement.	“Even when I saw a friend doing something wrong, I stayed quiet because I didn’t want them to feel embarrassed” (Case 13) “I felt pressure and didn’t dare to speak up when the teacher was already talking” (Case 7)
Theme 2: Stress as a Double-Edged Sword in Simulation	Sub-theme 2.1: Stress as a double-edged sword	Stress operated as a double-edged sword: it could heighten focus and prioritisation, yet could also tip into overload and reduce voice, particularly when in-scenario correction was experienced as abrupt.	“I froze at the beginning, but the nervousness made me remember the steps faster” (Case 11) “The pressure pushed me to think more quickly about what needed to come first” (Case 3) “When the professor corrected me sharply during the scenario, I lost confidence and stopped talking” (Case 14)
	Sub-theme 2.2: Role positioning as stress exposure	Active scenario participants experienced responsibility and time pressure that could narrow attention, whereas observers reported more cognitive space to scan the situation, compare responses, and rehearse alternatives.	“When I was the one doing it, I felt pressured and forgot small details” (Case 9) “As an observer, I could see everything and think about how I would handle it, but I didn’t always speak up” (Case 18)
	Sub-theme 2.3: Facilitation under pressure	Facilitation shaped how pressure was experienced: allowing the scenario to unfold and reserving step-by-step feedback for debrief supported learning from mistakes, while mid-scenario interruptions shifted attention towards fear of error.	“When the teacher let us finish first and then explained step by step, I felt clearer about what I had done right and wrong” (Case 13) “When the professor interrupted during the simulation, I lost focus on the patient and just worried about mistakes” (Case 14)
Theme 3: Learning Across Participation, Observation, and Debriefing	Sub-theme 3.1: Participation: learning through accountable action	Active participation made learning feel real; acting under pressure demanded decisions, prioritisation, and leadership, making lessons memorable.	“When I was inside, I felt pressured and nervous, but it made me think much faster about what to do first” (Case 11) “Being in the hot seat made me realise how important it was to stay calm and lead the group” (Case 16)
	Sub-theme 3.2: Observation: learning through distance and noticing	Observation offered a calmer, analytic vantage point, enabling students to see the whole scene, notice errors, and mentally rehearse how they would respond.	“As an observer, I could see what everyone was doing and think about how I would handle the situation” (Case 18) “Watching others made me realise mistakes and compare them with how I might respond” (Case 9)
	Sub-theme 3.3: Debriefing: integrating active participant and observer perspectives	Debriefing was where active scenario participant and observer perspectives were integrated; shared discussion helped students re-read the event, clarify rationales, and identify what they had missed.	“Afterwards, hearing both sides helped me realise what I had missed when I was inside” (Case 22) “Group discussion made me think again about my own actions” (Case 23)
Theme 4: Facilitation and Cultural Safety	Sub-theme 4.1: Calm guidance versus harsh correction	Students contrasted calm, invitational guidance with harsh correction; facilitator tone influenced whether they continued to engage or withdrew.	“If I met a professor who got angry, I felt pressured and didn’t dare to try anything” (Case 11) “When the teacher just listened and let us finish first, I felt safe to continue” (Case 17)
	Sub-theme 4.2: Debriefing as a constructive space	Students described debriefing as the point where learning became tangible; reviewing the scenario step by step enabled open reflection, normalised mistakes, and helped nervousness settle into confidence.	“When we reviewed it together, I could see what I did right and wrong, and I wasn’t afraid to admit mistakes” (Case 13)
	Sub-theme 4.3: Cultural safety intertwined with learning	Psychological safety was intertwined with cultural safety: kreng-jai and face concerns shaped whether students corrected peers or spoke up, making respectful facilitation central to participation.	“Even when I knew my friend had missed something, I didn’t correct them in front of others” (Case 13)

The Duality of Participation and Observation further showed that these positions supported different, but complementary, forms of learning. Students learned through direct action, through observation, and revisiting the same scenario during debriefing, where active scenario participant and observer perspectives were brought together.

In Facilitation and Cultural Safety, students consistently described how feedback was delivered as shaping whether they stayed engaged. When guidance was calm and appropriately timed, they tended to continue working through the scenario. By contrast, abrupt correction often made them hold back. These responses were closely linked to kreng-jai, where speaking up requires students to balance respect for hierarchy with the risk of causing discomfort to others.

Across the themes, simulation was understood not simply as a space for practising skills, but as a relational form of learning shaped by classroom hierarchy. Participation was not something automatically enabled by the design of simulation; rather, it took shape in the moment—through roles, facilitation, and the cultural expectations surrounding interaction.

### Theme 1: Entering a new space—navigating from classroom to simulation

For many Thai nursing students, the simulation room felt like a “third space”: no longer the familiarity of lectures, but not yet the ward. Their accounts clustered around three connected strands—pressure, realism, and silence—each shaping what it meant to enter this environment.

#### Sub-theme 1.1: The weight of pressure

Students described early simulation encounters as emotionally weighty, with stress exceeding what they typically felt in classroom learning. One student said, *“Every subject felt stressful… when I entered the simulation*,* the pressure was already there”* (Case 7). Intensity also shifted with role expectations. As the same participant noted, *“When I was the one inside the scenario*,* it was more stressful than when I was just observing”* (Case 7). Across these accounts, the simulation room carried an evaluative tension—felt in the body and in the moment—that students read as part of learning to “become” a nurse.

#### Sub-theme 1.2: Realism as rehearsal for practice

Alongside pressure, students valued simulation for how close it felt to practice. Several described it as a space where they could test themselves under near-clinical conditions, make mistakes, and then recalibrate. One student explained, *“It let me practice caring for patients on my own; it felt like I could really try and then see how to improve”* (Case 8). Another added, *“When I went in*,* it was as if I was already caring for a patient*,* not just studying in class”* (Case 12). What mattered here was not realism as a technical property alone, but realism as a learning cue—helping classroom knowledge move from being remembered to being enacted.

#### Sub-theme 1.3: Silence as a cultural stance

Students also described cultural norms that shaped participation inside the simulation space. Hesitation to speak up—especially to correct peers—was reported repeatedly. One student shared, *“Even when I saw a friend doing something wrong*,* I stayed quiet because I didn’t want them to feel embarrassed”* (Case 13). Another reflected on restraint during group interactions: *“I felt pressure and didn’t dare to speak up when the teacher was already talking”* (Case 7). Importantly, these silences were framed as relational work: showing kreng-jai, protecting face, and maintaining harmony, even when doing so limited immediate feedback or peer correction.

Overall, Theme 1 described simulation as a threshold—stressful yet formative, realistic yet socially bounded—where students practised skills while navigating respect, belonging, and emerging professional identity.

### Theme 2: Stress as a double-edged sword in simulation

Students described simulation as emotionally intense. Stress and nervousness were part of the learning texture—sometimes supportive, sometimes disruptive. What students learned also depended on where they were positioned in the session: being an active scenario participant carried a different kind of pressure from watching as an observer.

#### Sub-theme 2.1: Stress as a double-edged sword

Many accounts centred on tension in the room. For some, that intensity appeared to heighten focus and speed up recall. One participant reflected, *“I froze at the beginning*,* but the nervousness made me remember the steps faster”* (Case 11). Another noted, *“The pressure pushed me to think more quickly about what needed to come first”* (Case 3). At other times, stress tipped into overload—particularly when correction was experienced as abrupt. As one student put it, *“When the professor corrected me sharply during the scenario*,* I lost confidence and stopped talking”* (Case 14). Across these descriptions, stress functioned as a double-edged sword: it could support judgement and prioritisation, yet it could also narrow participation and voice.

#### Sub-theme 2.2: Role positioning as stress exposure

Students drew a clear contrast between being inside the scenario and observing from outside it. Active scenario participants spoke about responsibility, visibility, excitement, and pressure, whereas observers described having more distance from the immediate stress of action. One student explained, *“It was different. The person who actually went into the scenario felt excited and pressured and had to manage their own role. Observers could see the overall picture and notice what others missed more easily*,* but they did not feel the same excitement as those inside the situation”* (Case 24). These accounts show that role positioning shaped not only what students did during simulation, but also how exposed, accountable, or emotionally activated they felt.

#### Sub-theme 2.3: Facilitation under pressure

Students repeatedly linked their stress experience to how facilitation was enacted in the moment. When instructors allowed the scenario to unfold and offered structured feedback in the debrief afterwards, students described feeling supported and able to learn from mistakes: *“When the teacher let us finish first and then explained step by step*,* I felt clearer about what I had done right and wrong”* (Case 13). In contrast, mid-scenario interruptions could shift attention away from the patient and towards fear of error: *“When the professor interrupted during the simulation*,* I lost focus on the patient and just worried about mistakes”* (Case 14).

Overall, Theme 2 described how stress and role positioning intersected with facilitation style to shape whether pressure functioned as productive challenge or as a constraint on participation and confidence.

### Theme 3: Learning across participation, observation, and debriefing

Students described learning in simulation through two positions: being “in the hot seat” and watching from the outside. These were not portrayed as equivalent experiences. Each position offered different learning affordances, and students often made sense of the session by moving between these perspectives—especially when they returned to the event during debriefing.

#### Sub-theme 3.1: Participation: learning through accountable action

Being in an active scenario role was described as stressful, yet many students also framed it as the moment learning became real. Inside the scenario, they felt pressure to prioritise and act with accountability. As one student put it, *“When I was inside*,* I felt pressured and nervous*,* but it made me think much faster about what to do first”* (Case 11). Another linked this position to leadership and self-control: *“Being in the hot seat made me realise how important it was to stay calm and lead the group”* (Case 16). For these students, participation did not simply test knowledge; it demanded decisions, and acting under pressure made lessons harder to forget.

#### Sub-theme 3.2: Observation: learning through distance and noticing

Observers described a calmer experience, with more room to scan the situation and think through alternatives. One student explained, *“As an observer*,* I could see what everyone was doing and think about how I would handle the situation”* (Case 18). Another noted, *“Watching others made me realise mistakes and compare them with how I might respond”* (Case 9). Here, distance created perspective. Watching became a quiet form of rehearsal—an opportunity to notice patterns and anticipate responses that could be difficult to access when one is inside the action.

#### Sub-theme 3.3: Debriefing: integrating active participant and observer perspectives

Students repeatedly pointed to debriefing as the point where these two viewpoints came together. Several described that understanding shifted after hearing how active scenario participants and observers interpreted the same scene. One participant said, *“Afterwards*,* hearing both sides helped me realise what I had missed when I was inside”* (Case 22). Another reflected, *“Group discussion made me think again about my own actions”* (Case 23). In these accounts, debriefing was where experience was re-read: what felt chaotic in the moment could be reorganised into clearer priorities, rationales, and alternatives.

Overall, Theme 3 described learning as movement across roles—doing, watching, and revisiting the event together.

### Theme 4: Facilitation and cultural safety

Unlike the preceding themes, this theme did not refer to a single phase of the simulation flow. It operated as a cross-cutting condition, shaping how students experienced pressure at entry, role demands during the scenario, and reflection during debriefing. The theme is presented last not because it occurred last, but because its influence threaded through what came before.

Students rarely spoke about facilitation as background. The facilitator was “felt” in the room through tone, timing, and the way feedback entered the scenario. What students noticed most was whether guidance opened space to try, or whether it closed that space down and made silence feel safer.

#### Sub-theme 4.1: Calm guidance versus harsh correction

Participants contrasted supportive guidance with moments when correction felt sharp. One student recalled, *“If I met a professor who got angry*,* I felt pressured and didn’t dare to try anything”* (Case 11). Others described the opposite effect when teachers stayed calm and allowed them to continue even when they faltered. As a fourth-year student explained, *“When the teacher just listened and let us finish first*,* I felt safe to continue”* (Case 17). Across these accounts, the way feedback was delivered shaped whether students stayed engaged or withdrew.

#### Sub-theme 4.2: Debriefing as a constructive space

For many students, the debrief was where learning became tangible. They valued reviewing the scenario step by step, not only to check correctness but also to speak more openly about what they were thinking and feeling. One participant said, *“When we reviewed it together*,* I could see what I did right and wrong*,* and I wasn’t afraid to admit mistakes”* (Case 13). Others described how a calm debrief helped nervousness settle into confidence. In this way, debriefing was not merely a wrap-up; its atmosphere shaped whether mistakes were experienced as usable lessons or as personal failure.

#### Sub-theme 4.3: Cultural safety intertwined with learning

Facilitation also interacted with cultural expectations around speaking, respect, and protecting others from embarrassment. Silence in discussion was often described as relational rather than disengaged. One student admitted, *“Even when I knew my friend had missed something*,* I didn’t correct them in front of others”* (Case 13). For these learners, kreng-jai—the impulse to avoid causing discomfort—guided how they responded to both peers and teachers. Students’ accounts consistently linked psychological safety with cultural safety: when both were present, students felt more able to contribute; when either was threatened, withholding voice became the safer option.

Overall, Theme 4 described facilitation as more than a teaching technique: calm feedback and respectful debriefing supported participation, whereas harsh correction reduced voice and confidence, with these effects intensified by cultural expectations around hierarchy and face.

## Discussion

This study offers a culturally situated account of simulation-based learning (SBL) in Thai undergraduate nursing education. Students often described SBL as more than rehearsal. For many, it was experienced as an early moment of professional entry: highly visible, emotionally charged, and shaped by familiar classroom norms. This reading sits alongside broader syntheses showing that SBL can support competence development, confidence, and preparedness across diverse curricular approaches [1, 10]. What our participants emphasised, however, was how real the nurse role felt in simulation, sometimes earlier than comparable opportunities in practice. Stress, observer learning, and fidelity are well-established topics in simulation scholarship. What this study adds is not the identification of entirely new phenomena, but an account of how these familiar features were experienced and mediated in a context where hierarchy, face, and kreng-jai shaped what students felt able to say and do. The contribution therefore lies less in introducing new constructs than in showing how established constructs may operate differently when relational and cultural conditions are foregrounded. Where early clinical exposure is perceived as limited or delayed, simulation may function as a liminal space in which theoretical knowledge is tested against the situated demands of patient care. This interpretation aligns with sociocultural perspectives that view learning as participation within communities of practice rather than as the simple acquisition of decontextualised skills [19].

**Fig. 1:**
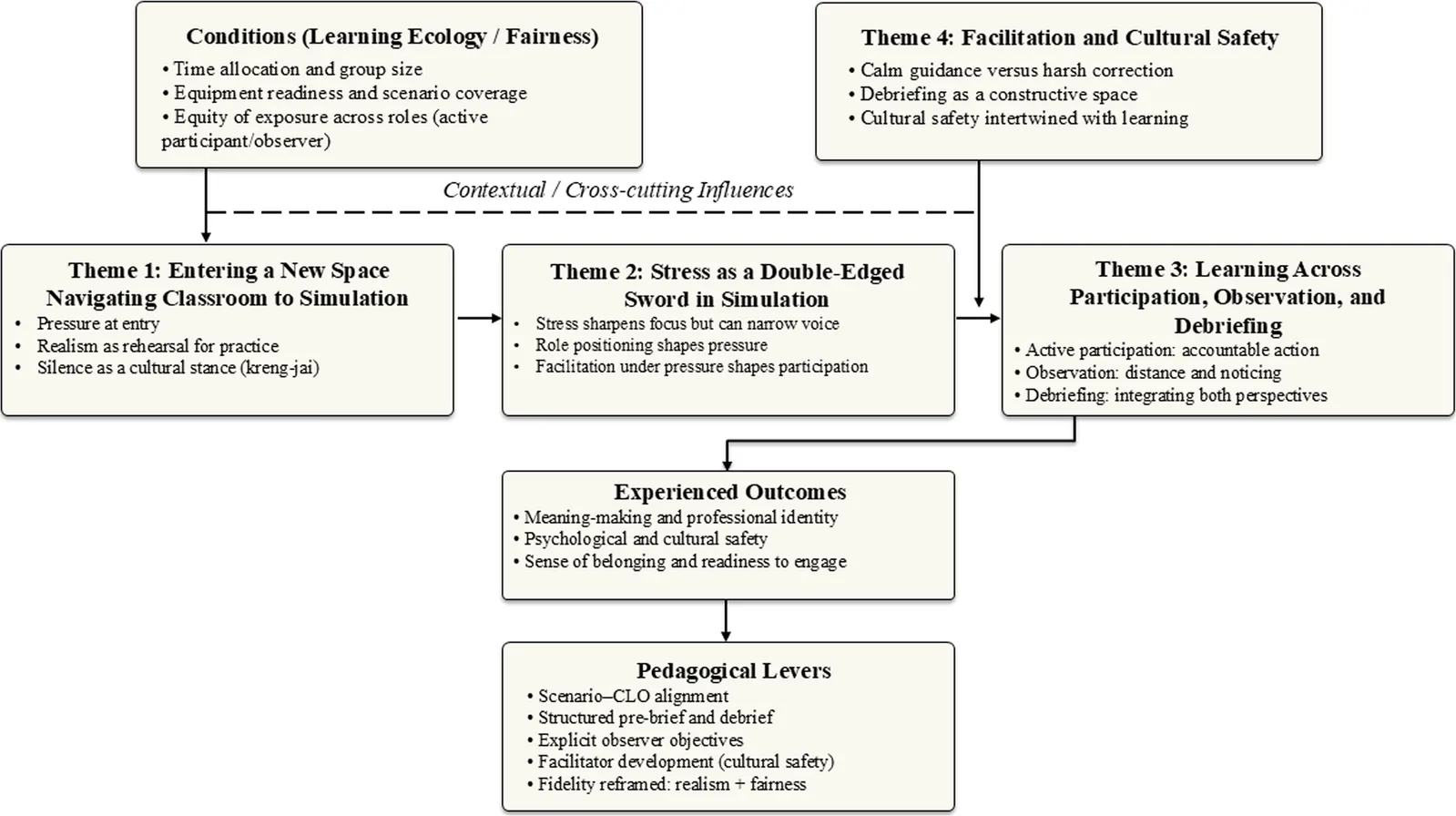
Thematic map of Thai nursing students' experiences of simulation-based learning. Note. The figure summarises patterned meanings developed through reflexive thematic analysis. Facilitation and cultural safety are shown as cross-cutting conditions across the simulation flow. The figure should not be read as a causal model

To integrate the findings, Fig. 1 presents a thematic map of how Thai nursing students experienced simulation-based learning as a pedagogical flow. Students entered the simulation space with heightened pressure and a sense of realism (Theme 1), which shaped stress and role positioning during the scenario (Theme 2). Learning then unfolded through complementary active participant and observer perspectives (Theme 3), with understanding consolidated through debriefing as shared reflection. Across this flow, the cross-cutting theme of facilitation and cultural safety (Theme 4) shaped how pressure, feedback, and silence were experienced at multiple points. The figure is intended as an analytic summary of patterned meanings rather than a causal model. It highlights how contextual conditions, role positioning, and facilitation intersected to shape meaning-making, perceived safety, and willingness to participate.

Across interviews, kreng-jai emerged as a salient mediator of participation. This resonates with Thai evidence showing how hierarchy, kreng-jai, and feedback norms shape what is said, to whom, and when [2]. It is also consistent with work describing “silent participation” as culturally intelligible rather than disengaged [11]. Our findings extend this discussion by suggesting that facilitator tone and timing can either widen or narrow the space for student voice. When facilitation felt calm and non-punitive, deference sometimes shifted towards respectful engagement, allowing students to ask questions and acknowledge uncertainty without embarrassment. In contrast, harsh or abrupt correction could prompt withdrawal that appeared less related to knowledge deficits than to relational risk-management in the face of hierarchy [2]. These patterns were not identical for every participant, but they recurred often enough to shape how debriefing was experienced. In this reading, psychological safety was not a fixed background condition. It was interactionally produced, and could be strengthened or undermined through how feedback was timed, voiced, and socially positioned.

These findings underline the practical importance of culturally responsive facilitation and debriefing, particularly given that much of the dominant debriefing discourse has been developed in Western educational traditions and may require culture-sensitive adaptation in high power-distance Asian settings [16, 30]. Debriefing approaches such as advocacy-inquiry often rely on learners being willing to voice their reasoning when invited to do so. This assumption fits educational cultures characterised by lower power distance and more individualist orientations, where open disagreement with instructors is more readily tolerated [15]. In collectivist settings, different calculations may apply. Learners may prioritise group harmony and face preservation over individual expression, particularly when speaking risks exposing a peer’s error or implying criticism of a senior. Our participants described exactly this pattern: holding back not because they lacked insight, but because voicing it felt relationally risky. These dynamics do not invalidate Western debriefing frameworks, but they do suggest that prompts designed to elicit verbal reflection may need to be adapted, or supplemented with alternative participation structures, when used in high power-distance Asian contexts.

The contrast between learning through active participation and learning through observation also offers an important insight. Students in-role described learning through performance under pressure, whereas observers more often described a reflective stance, noticing patterns, errors, and alternative actions from a distance. This is consistent with social learning accounts of vicarious learning, in which learning can occur through observing others and the consequences of action [4]. Our findings add a practical refinement to this account: observational learning appeared most useful when it was intentionally structured, for example through guided prompts or focused questions, rather than left as passive watching. This is especially relevant in settings where opportunities to perform are constrained by time, group size, or available equipment. Under such conditions, the observer role may be better understood as an active learning position rather than a secondary one [5].

The findings also point to a tension between the visible infrastructure of simulation and the less visible pedagogical conditions that students described as shaping learning, including facilitator practice, debriefing climate, and psychological safety. Rather than equating fidelity with equipment alone, our data support a broader understanding of simulation quality as a socially organised learning practice. This aligns with theoretical arguments that simulation is not only a technical set-up, but also a form of social practice in which interactional conditions matter [13]. Read in this way, simulation quality extends beyond physical realism to include psychological fidelity, that is, the extent to which learners experience the scenario as meaningful, believable, and emotionally safe [9]. From this perspective, SBL is less a technological showcase than a relational pedagogy, strengthened through facilitation that is both psychologically safe and culturally attuned.

Students’ accounts of facilitator tone and correction suggest that psychological safety was created not only during debriefing. It had to be built from the beginning of the simulation activity. Prebriefing may help in this process by explaining not only equipment, roles, and objectives, but also what students should expect if they feel uncertain, make a mistake, or receive feedback during the scenario [28]. In learning settings where teacher authority is strongly felt, public correction or open admission of uncertainty may be experienced as a threat to face, rather than simply as part of learning [14, 17]. Debriefing should continue this sense of safety. A structured model such as GAS or Plus-Delta may be useful because it starts with learners’ descriptions before moving to performance analysis. Learners are first invited to describe what happened, how they felt, and what they noticed during the scenario [21, 23]. The analysis phase can then begin from the learner’s own account of the event: what they noticed, how they understood the situation, and what they were trying to do. This makes correction less abrupt because students have a chance to explain their thinking before the facilitator discusses what could be improved. In this study, culturally responsive facilitation shaped whether students experienced debriefing as a safe space for participation and reflection, or as a situation in which their mistakes were exposed to others.

### Policy and practice implications

For many participants, simulation was not experienced as a peripheral exercise but as an early—and highly visible—entry point into patient care roles. Current accreditation guidance tends to specify infrastructural requirements, such as facilities, mannequins, and equipment, more explicitly than pedagogical conditions such as facilitator preparation, structured debriefing, and learner psychological safety.

Several practical implications follow. First, the observer role could be formalised. Participants described observation as cognitively useful; programmes could therefore treat observers as active learners by assigning explicit objectives and using short observer worksheets or checklists to guide what they notice during the scenario and later discuss in debriefing. Second, debriefing warrants greater emphasis. Although infrastructural requirements are often more explicitly specified, participants’ accounts suggest that culturally attuned debriefing can enable reflection without fear of embarrassment. In practice, this may involve minimising abrupt in-scenario correction, allowing learners to complete key actions before feedback, and using invitational prompts that support explanation without unnecessary exposure. Finally, participants framed fidelity not only as physical realism but also as fairness—whether opportunities were equitable across groups. Standards that address group size, time allocation, role rotation, and equipment readiness may therefore be as consequential as equipment investment for sustaining trust in simulation.

### Limitations and future directions

This study has several limitations. First, it was conducted in a single institution, which supported contextual depth but constrained breadth. Second, although the qualitative findings spoke to constructs also examined quantitatively, the analysis was not designed to estimate magnitude or test associations; rather, it explored how students made sense of these experiences and the conditions under which they shifted. Third, although stress inoculation and dual-process ideas informed the interpretive lens, the underlying mechanisms were not directly tested, and cognitive load, physiological markers, and longer-term transfer were not assessed. Fourth, because third- and fourth-year students may differ in clinical maturity and prior exposure to SBL, the findings should not be read as representing a single homogeneous learner experience. Finally, comparative discussion drew on a still limited body of evidence from ASEAN settings, and the uneven regional literature constrains broader transferability claims. Future work could test specific pedagogical levers, such as structured debriefing and observer task design, across multiple sites and programme years.

## Conclusion

This study offers a culturally grounded account in which simulation was experienced less as skill reinforcement and more as an early form of professional initiation. In contexts where clinical exposure is limited or delayed, simulation may function not as repetition of prior knowledge but as an initial encounter with what it means to act as a nurse. Students described it as a bounded space in which errors could be surfaced and discussed without risk to patients, even though participation within that space remained shaped by relational and cultural risk.

Participation was shaped by more than scenario design alone. Cultural norms around hierarchy and silence affected how willing students were to speak, question decisions, or voice uncertainty aloud. Facilitation also influenced whether students remained engaged. When feedback was delivered calmly and at appropriate moments, students were more likely to continue participating. In contrast, abrupt correction often led to withdrawal, particularly when the debriefing environment was experienced as exposing rather than safe.

The findings point to a potential gap between infrastructure-focused standards and the conditions students identified as formative. Accreditation criteria tend to emphasise equipment and physical space, whereas students emphasised relational and pedagogical conditions. Greater attention to debriefing quality, observer role design, and equitable opportunities to participate may bring simulation provision into closer alignment with current evidence on learning.

The extent to which these patterns extend to other settings remains uncertain. The findings may nevertheless be relevant for educators working in other collectivist or hierarchical contexts, where voice, status, and relational norms shape simulation outcomes in ways that cannot be addressed through equipment alone.

## Supplementary Information

Supplementary Material 1.

## Data Availability

The interview transcripts are not publicly available due to confidentiality and the risk of participant identification. De-identified excerpts may be available from the corresponding author upon reasonable request and subject to ethics approval/conditions.

## References

[1] Alharbi A, Nurfianti A, Mullen RF, McClure JD, Miller WH. The effectiveness of simulation-based learning (SBL) on students’ knowledge and skills in nursing programs: a systematic review. BMC Med Educ. 2024;24(1):1099. 10.1186/s12909-024-06080-z. PMC11459713 39375684

[2] Areemit RS, Cooper CM, Wirasorn K, Paopongsawan P, Panthongviriyakul C, Ramani S. Hierarchy, Kreng Jai and Feedback: A Grounded Theory Study Exploring Perspectives of Clinical Faculty and Medical Students in Thailand. Teach Learn Med. 2021;33(3):235–44. 10.1080/10401334.2020.1813584. 33023318

[3] Arpanantikul M, Pratoomwan A. Clinical Learning Experiences of Second-Year Thai Nursing Students: A Phenomenological Study. Pac Rim Int J Nurs Res. 2017;21:121–34.

[4] Bandura A. Self-efficacy: Toward a unifying theory of behavioral change. Adv Behav Res Therapy. 1978;1(4):139–61. 10.1016/0146-6402(78)90002-4. 847061

[5] Bø B, Madangi BP, Ralaitafika H, Ersdal HL, Tjoflåt I. Nursing students’ experiences with simulation-based education as a pedagogic method in low-resource settings: A mixed-method study. J Clin Nurs. 2022;31(9–10):1362–76. 10.1111/jocn.15996. 34423486

[6] Braun V, Clarke V. Reflecting on reflexive thematic analysis. Qualitative Res Sport Exerc Health. 2019;11(4):589–97. 10.1080/2159676X.2019.1628806.

[7] Braun V, Clarke V. To saturate or not to saturate? Questioning data saturation as a useful concept for thematic analysis and sample-size rationales. Qualitative Res Sport Exerc Health. 2021;13(2):201–16. 10.1080/2159676X.2019.1704846.

[8] Braun V, Clarke V. Thematic analysis: a practical guide. London: SAGE Publications Ltd; 2022.

[9] Burford B, Grieg P, Kelleher M, Merriman C, Platt A, Richards E, Davidson N, Vance G. Interprofessional simulation as an enhancement of psychological fidelity: A focus group study of medical and nursing students. Health Sci Rep. 2023;6(5):e1237. 10.1002/hsr2.1237. PMC10154877 37152225

[10] Cant RP, Cooper SJ. Use of simulation-based learning in undergraduate nurse education: An umbrella systematic review. Nurse Educ Today. 2017;49:63–71. 10.1016/j.nedt.2016.11.015. 27902949

[11] Chung JHJ. We Participate, Silently: Explicating Thai University Students’ Perceptions of Their Classroom Participation and Communication. Qualitative Res Educ. 2021;10(1):62–87. 10.17583/qre.2021.7159.

[12] Diaz-Navarro C, Armstrong R, Charnetski M, Freeman KJ, Koh S, Reedy G, Smitten J, Ingrassia PL, Matos FM, Issenberg B. Global consensus statement on simulation-based practice in healthcare. Adv Simul. 2024;9(1):19. 10.1186/s41077-024-00288-1. PMC11106913 38769577

[13] Dieckmann P, Gaba D, Rall M. Deepening the theoretical foundations of patient simulation as social practice. Simul Healthc. 2007;2(3):183–93. 10.1097/SIH.0b013e3180f637f5. 19088622

[14] Edmondson A. Psychological Safety and Learning Behavior in Work Teams. Adm Sci Q. 1999;44(2):350–83. 10.2307/2666999.

[15] Hofstede G. Culture’s consequences: comparing values, behaviors, institutions, and organizations across nations. 2nd ed. Thousand Oaks, CA: SAGE Publications; 2001.

[16] Hyun Soo C, Peter D, Saul Barry I. It is time to consider cultural differences in debriefing. Simul Healthc. 2013;8(3):166–70. 10.1097/SIH.0b013e318291d9ef. 23702587

[17] Kolbe M, Eppich W, Rudolph J, Meguerdichian M, Catena H, Cripps A, Grant V, Cheng A. Managing psychological safety in debriefings: a dynamic balancing act. BMJ Simul Technol Enhanc Learn. 2020;6(3):164–71. 10.1136/bmjstel-2019-000470. PMC8936758 35518370

[18] Kuesakul K, Nuampa S, Pungbangkadee R, Ramjan L, Ratinthorn A. Evaluation of antenatal simulation-based learning on satisfaction and self-confidence levels among Thai undergraduate nursing students during the COVID-19 pandemic: a mixed-method study. BMC Nurs. 2024;23(1):161. 10.1186/s12912-024-01824-0. PMC10916063 38448962

[19] Lave J, Wenger E. Situated learning: Legitimate peripheral participation. Cambridge University Press; 1991. 10.1017/CBO9780511815355.

[20] Malterud K, Siersma VD, Guassora AD. Sample Size in Qualitative Interview Studies: Guided by Information Power. Qual Health Res. 2016;26(13):1753–60. 10.1177/1049732315617444. 26613970

[21] Mitchell A. Debriefing after a simulation activity: using the Plus Delta debrief model. Br J Nurs. 2025;34(22):1147–8. 10.12968/bjon.2025.0257. 41355568

[22] Olaussen C, Heggdal K, Tvedt CR. Elements in scenario-based simulation associated with nursing students’ self-confidence and satisfaction: A cross-sectional study. Nurs Open. 2020;7(1):170–9. 10.1002/nop2.375. PMC6917966 31871700

[23] Phrampus PE, O’Donnell JM. Debriefing Using a Structured and Supported Approach. In A. I. Levine, S. DeMaria, A. D. Schwartz, & A. J. Sim, editors, The Comprehensive Textbook of Healthcare Simulation. 2013:73–84. Springer New York. 10.1007/978-1-4614-5993-4_6.

[24] Polit DF, Beck CT. Nursing research: generating and assessing evidence for nursing practice. 10th ed. Philadelphia, PA: Wolters Kluwer Health; 2017.

[25] Rudolph JW, Simon R, Dufresne RL, Raemer DB. There’s No Such Thing as Nonjudgmental Debriefing: A Theory and Method for Debriefing with Good Judgment. Simul Healthc. 2006;1(1). 10.1097/01266021-200600110-00006. 19088574

[26] Skedsmo K, Bingen HM, Hofsø K, Steindal SA, Hagelin CL, Hilderson D, Nes AAG, Smis D, Stenseth HV, Olaussen C. Postgraduate nursing students’ experiences with simulation-based learning in palliative care education: A qualitative study. Nurse Educ Pract. 2023;73:103832. 10.1016/j.nepr.2023.103832. 37948917

[27] Squires A. Methodological challenges in cross-language qualitative research: A research review. Int J Nurs Stud. 2009;46(2):277–87. 10.1016/j.ijnurstu.2008.08.006. PMC2784094 18789799

[28] Standards Committee INACSL, Watts PI, McDermott DS, Alinier G, Charnetski M, Ludlow J, Horsley E, Meakim C, Nawathe PA. Healthcare Simulation Standards of Best Practice: Simulation Design. Clin Simul Nurs. 2021;58:14–21. 10.1016/j.ecns.2021.08.009.

[29] Temple B, Young A. Qualitative Research and Translation Dilemmas. Qualitative Res. 2004;4(2):161–78. 10.1177/1468794104044430.

[30] Ulmer FF, Sharara-Chami R, Lakissian Z, Stocker M, Scott E, Dieckmann P. Cultural prototypes and differences in simulation debriefing. Simul Healthc. 2018;13(4):239–46. 10.1097/SIH.0000000000000320. 29672469

[31] van Graan AC, Williams MJS, Koen MP. Professional nurses’ understanding of clinical judgement: A contextual inquiry. Health SA Gesondheid. 2016;21:280–93. 10.1016/j.hsag.2016.04.001.

[32] van Nes F, Abma T, Jonsson H, Deeg D. Language differences in qualitative research: is meaning lost in translation? Eur J Ageing. 2010;7(4):313–6. 10.1007/s10433-010-0168-y. PMC2995873 21212820

[33] Xuto P, Prasitwattanaseree P, Chaiboonruang T, Khiaokham L, Nimarangkul K, Bressington D, Miyata C. Developing clinical leadership through high-fidelity obstetric simulation: A multimethods study in Thai nursing students. Clin Simul Nurs. 2025;102:101720. 10.1016/j.ecns.2025.101720.

