# Supplementary material for: Thai nursing students’ experiences of stress, role positioning, and facilitation tone in simulation-based learning: implications for culturally responsive facilitation and debriefing
Source: Adv Simul (Lond). 2026 Jul 18;11:75. doi: 10.1186/s41077-026-00472-5 (PMC13670541; doi:10.1186/s41077-026-00472-5)
Supplement: Supplementary file 1 [file 41077_2026_472_MOESM1_ESM.pdf]

Supplementary File 1. Semi-Structured Interview Guide: English Translation and Original Thai Version

Part A. English Translation of the Semi-Structured Interview Guide

This supplementary file includes the English translation of the semi-structured interview guide followed by the original Thai version used for data collection.

Original study title: Satisfaction with Simulation-Based Learning, Clinical Judgement, and Confidence in Clinical Skills as Perceived by Nursing Students

This document presents an English translation of the original Thai semi-structured interview guide used for data collection. The guide was developed for the qualitative component of a broader study examining nursing students' experiences of simulation-based learning, with attention to satisfaction, clinical judgement, and confidence in clinical skills.

The interview began with an introduction to the study purpose, an explanation of confidentiality, and permission to audio-record the interview. Participants were informed that the interview would take approximately one hour and that they could decline to answer any question or withdraw from the interview at any time.

Note. *The questions presented in this interview guide served as flexible prompts to guide data collection. During the interviews, the wording and sequence of questions could be adapted according to the conversational flow and participants' responses. Additional probes were used when necessary to clarify meanings, explore emerging issues, and follow up on relevant or unexpected insights identified during the interviews.*

Opening Questions

1. How are you feeling today?
2. What year of study are you currently in?
3. What was your previous grade point average?
4. How would you describe your general life or learning experiences at this stage?

Experiences with Simulation-Based Learning

Main question

1. Could you describe your experience of learning through simulation-based learning?

Probing questions

1. What did you learn from the simulation-based learning session?
2. How would you describe the learning atmosphere during the simulation?

Satisfaction with Simulation-Based Learning

Main question

1. How satisfied were you with simulation-based learning?

Probing questions

1. What aspects of simulation-based learning made you feel satisfied?
2. What aspects of simulation-based learning made you feel dissatisfied or less satisfied?
3. Which components of simulation-based learning did you like most?
4. What aspects of simulation-based learning should be improved?
5. In your view, how did simulation-based learning help develop your nursing skills?
6. From your perspective, what similarities or differences were there between learning in the skills laboratory and learning through simulation-based learning?
7. From your perspective, how did simulation-based learning differ from traditional learning approaches?
8. How did you feel when taking the role of a learner in the simulation compared with taking the role of a nurse in a clinical practicum? In what ways were these experiences similar or different?
9. How did the process of simulation-based learning help you reflect on your own role in the scenario and your future professional role as a nurse?

Clinical Judgement

Main question

1. How did simulation-based learning affect your clinical judgement as a nursing student?

Probing questions

1. How were you able to apply theoretical knowledge to practice during simulation-based learning?
2. After participating in simulation-based learning, how did your ability to observe and identify changes in a patient's condition change? Why?
3. After participating in simulation-based learning, how did your ability to interpret patients' symptoms and clinical manifestations change? Why?
4. After participating in simulation-based learning, how did your ability to respond promptly to changes in a patient's condition and nursing care needs change? Why?

5. How did simulation-based learning promote your clinical decision-making as a nursing student? Why?
6. In what ways did simulation-based learning help develop your clinical judgement?

Confidence in Clinical Skills

Main question

1. After participating in simulation-based learning, was your confidence sufficient for practising in real clinical settings? Why?

Probing questions

1. After participating in simulation-based learning, how confident were you in assessing a patient's signs and symptoms and identifying abnormal findings?
2. How did simulation-based learning help you acquire the knowledge needed for clinical nursing practice?
3. After participating in simulation-based learning, how confident were you in managing incidents or unexpected situations that may occur during patient care?
4. After participating in simulation-based learning, how confident were you in applying the knowledge gained from the simulated scenario to real patient care?

Closing Questions

1. Is there anything else you would like to add about your experience of simulation-based learning?
2. Is there anything you would like to clarify, expand on, or emphasise before we end the interview?

At the end of the interview, the interviewer thanked the participant and provided contact information in case the participant had concerns, experienced distress, or wished to withdraw from the study.

Part B. Original Thai Semi-Structured Interview Guide

ส่วนที่ 5 แบบสัมภาษณ์กึ่งโครงสร้าง (ส่วนนี้ผู้วิจัยเป็นผู้บันทึก)

โครงการวิจัยเรื่อง ความพึงพอใจต่อการจัดการเรียนการสอนด้วยสถานการณ์จำลองเสมือนจริง ความสามารถในการตัดสินทางคลินิกและความมั่นใจในทักษะทางคลินิก ตามการรับรู้ของนิสิตพยาบาล

รหัสประจำตัวผู้เข้าร่วม:________ เพศ ชาย / หญิง วันที่ ____/____/____

สถานที่___________________ ผู้สัมภาษณ์______________ ชื่อไฟล์เสียง___________

วันที่สัมภาษณ์...............................

เวลาเริ่ม___:___

ขั้นเริ่มต้น

เกริ่นนำและชี้แจงวัตถุประสงค์

สวัสดีครับ/คะ ขอบคุณนิสิตที่มาร่วมพูดคุยกันในวันนี้ตอนนี้พวกเรากำลังดำเนินการโครงการวิจัยเกี่ยวกับความพึงพอใจต่อการจัดการเรียนการสอนด้วยสถานการณ์จำลองเสมือนจริง ความสามารถในการตัดสินทางคลินิกและความมั่นใจในทักษะทางคลินิก พวกเรามั่นใจว่า ข้อมูลจากนิสิต มีความสำคัญเป็นอย่างมาก ในมุมมองต่อการจัดการเรียนการสอนด้วยสถานการณ์จำลองเสมือนจริง.

กระบวนการสัมภาษณ์

การสนทนาจะเริ่มจากการสอบถามข้อมูลเบื้องต้น ประสบการณ์ที่ผ่านมา หรือความคิดเห็นของน้องเกี่ยวกับ การจัดการเรียนการสอนด้วยสถานการณ์จำลองเสมือนจริง คำตอบที่ตอบนั้นจะไม่มีการตัดสินว่าถูกหรือผิด

การขออนุญาต

การสนทนาจะใช้เวลาประมาณ ไม่เกิน 1 ชั่วโมง และ เพื่อให้ได้ข้อมูลครบถ้วน ในระหว่างการสนทนาจะต้องมีการบันทึกเสียง ซึ่งจะมีเพียงแค่ทีมวิจัยเท่านั้นที่จะสามารถฟังเสียงที่ทำการบันทึกไว้ได้

การพิทักษ์สิทธิ์

คำถามในระหว่างการสนทนาจะประกอบไปด้วย 4 ส่วน คำถามที่ไม่อยากจะตอบ นิสิตไม่จำเป็นต้องตอบคำถามเหล่านั้น รวมถึงเราสามารถยุติการสนทนาได้ทุกเวลา ข้อมูลทั้งหมดจะถูกเก็บเป็นความลับ โดยจะมีเพียงแค่นักวิจัยเท่านั้นที่จะสามารถเข้าถึงข้อมูลได้ โครงการวิจัยนี้ได้รับการอนุมัติจากคณะกรรมการพิจารณาจริยธรรมการวิจัยในมนุษย์ มหาวิทยาลัยบูรพา

มีข้อสงสัยเกี่ยวกับข้อมูลที่ได้อธิบายเบื้องต้นหรือไม่ครับ/คะ ถ้าไม่มีสามารถลงลายมือชื่อในเอกสารแบบแสดงความยินยอมเพื่อเข้าร่วมโครงการวิจัยได้เลย

หัวข้อหลัก	คำถามหลักและคำถามรอง
**ข้อมูลทั่วไป**	**คำถามหลัก**<br>ขอสอบถามเกี่ยวกับข้อมูลส่วนตัวหน่อยได้ไหม

BUU-IRB Approved
14 Jun 2024

หัวข้อหลัก	คำถามหลักและคำถามรอง
	**คำถามรอง**<br>• อายุเท่าไหร่แล้วตอนนี้<br>• เรียนอยู่ชั้นปีไหน<br>• เกรดเฉลี่ยเป็นอย่างไรบ้าง<br>• ชีวิตในช่วงนี้เป็นอย่างไรบ้าง
**ประสบการณ์การเรียน** ด้วยสถานการณ์จำลองเสมือนจริง	**คำถามหลัก**<br>ต่อไปนี้จะถามเกี่ยวกับประสบการณ์**การเรียน**ด้วยสถานการณ์จำลองเสมือนจริง<br>• ที่ผ่านมาเรียนด้วย SBL ในวิชาอะไรบ้าง เป็นอย่างไร<br>• บรรยากาศการเรียนเป็นอย่างไร
ความพึงพอใจต่อการจัดการเรียนการสอน	**คำถามหลัก**<br>ความพึงพอใจต่อการจัดการเรียนการสอน SBL เป็นอย่างไร<br>**คำถามรอง**<br>• นิสิตรู้สึกอย่างไร ที่คณะจัดการเรียนการสอนด้วย SBL<br>• นิสิตมีความพึงพอใจต่อการจัดการเรียนการสอนด้วย simulation ในระดับใด (อธิบายระดับและความหมาย และให้นิสิตเลือกระดับ)<br>• อะไรคือจุดเด่นของการจัดการเรียนการสอนด้วย simulation<br>• อะไรคือสิ่งที่ควรปรับปรุงในการจัดการเรียนการสอนด้วย simulation<br>• นิสิตคิดว่าการจัดการเรียนการสอนด้วย simulation ช่วยพัฒนาทักษะอะไรแก่นิสิตบ้าง<br>• ในมุมมองของนิสิต ความเหมือนหรือความต่างของ การเรียนในห้องปฏิบัติกับการเรียนการสอนด้วย SBL เป็นอย่างไร<br>• ในมุมมองของนิสิตการเรียนด้วย SBL ทำให้เกิดความแตกต่างจากการเรียนแบบเดิมอย่างไร<br>• ในมุมมองของนิสิต นิสิตรู้สึกว่าการเข้าเป็นผู้เรียนในสถานการ์จำลองเสมือนจริงกับการเป็นผู้สังเกตการณ์ มีความแตกต่างกันอย่างไร<br>• กระบวนการจัดการเรียนการสอนด้วย SBL สามารถช่วยให้นิสิตที่เข้าไปแสดงบทบาทในสถานการณ์และผู้สังเกตการบรรลุตามวัตถุประสงค์รายวิชา หรือไม่อย่างไร
ความสามารถในการตัดสินทางคลินิก	**คำถามหลัก**<br>การเรียนด้วย SBL ช่วยพัฒนาความสามารถในการตัดสินทางคลินิกของนิสิตพยาบาลอย่างไร

BUU IRB Approved
14 Jun 2024

หัวข้อหลัก	คำถามหลักและคำถามรอง
	**คำถามรอง**<br>• มีการนำความรู้จากภาคทฤษฎี ไปประยุกต์ใช้ในการเรียนด้วย SBL อย่างไรบ้าง<br>• นิสิตคิดว่าหลังจากเรียน SBL แล้ว นิสิตสามารถสังเกตเห็นความเปลี่ยนแปลงที่เกิดขึ้นกับผู้รับบริการมีมากน้อยเพียงใด และเพราะอะไร<br>• นิสิตคิดว่า หลังจากเรียน SBL แล้ว นิสิตสามารถแปลความหมายของอาการและอาการแสดงที่เกิดขึ้นกับผู้รับบริการ ได้หรือไม่ และเพราะอะไร<br>• นิสิตคิดว่า หลังจากเรียน SBL แล้ว นิสิตสามารถตอบสนองต่อการอาการที่เปลี่ยนแปลง และความต้องการด้านการพยาบาลของผู้รับบริการได้ทันท่วงทีหรือไม่ เพราะอะไร<br>• การเรียนด้วย SBL ทำให้นิสิตมีความสามารถในการตัดสินทางคลินิกของนิสิตได้ดีขึ้นหรือไม่ อย่างไร<br>• การเรียนด้วย SBL ช่วยให้นิสิตพัฒนาตนเองอย่างไรบ้าง
ความมั่นใจในทักษะทางคลินิกของนิสิตพยาบาล	**คำถามหลัก**<br>• ภายหลังจากที่เรียนด้วย SBL มีความมั่นใจมากน้อยเพียงใดในการฝึกประสบการณ์วิชาชีพจริงบนหอผู้ป่วย และเพราะอะไร<br>**คำถามรอง**<br>• หลังจากเรียนด้วย SBL ความมั่นใจในความสามารถในการประเมินอาการอาการแสดงของโรค และความผิดปกติของผู้ป่วยเป็นอย่างไร<br>• การเรียนด้วย SBL ช่วยให้นิสิตได้รับความรู้ที่จำเป็นในการปฏิบัติการพยาบาลทางคลินิกหรือไม่อย่างไร<br>• หลังจากเรียน SBL ความมั่นใจในความสามารถของตนเองเพื่อการจัดการกับเหตุการณ์ที่ไม่คาดคิดในการปฏิบัติการพยาบาลแก่ผู้ป่วยจริงเป็นอย่างไร<br>• หลังจากเรียน SBL ความมั่นใจที่จะนำความรู้ที่ได้จากการเรียนในสถานการณ์จำลองเสมือนจริงมาแก้ไขปัญหาผู้ป่วยในการปฏิบัติการพยาบาลจริงเป็นอย่างไร

ขั้นสรุป

นิสิตมีข้อมูลอะไรที่จะเล่าให้ฟังเพิ่มเติมอีกไหมในประเด็นเกี่ยวกับการเรียนด้วยสถานการจำลองเสมือนจริง

- สรุปวันนี้เราคุยกันเรื่อง
- กล่าวขอบคุณ

BUU-IRB Approved
14 Jun 2024

- ให้ข้อมูลเกี่ยวกับการติดต่อกลับหากมีประเด็นสงสัยเรื่องข้อมูล หรือต้องการจะบอกเลิกการเข้าร่วมการศึกษา

เวลาสิ้นสุด___:___

หมายเหตุ ข้อคำถามเป็นแนวทางในการเก็บรวมรวมข้อมูล ทั้งนี้ในระหว่างการเก็บรวมรวมข้อมูลอาจจะมีการปรับเปลี่ยนการใช้คำ หรือถามเจาะลึกในประเด็นต่าง ๆ ข้อค้นพบที่น่าสนใจ เพิ่มเติมได้

สรุปรายละเอียดการสัมภาษณ์โดยย่อ (ส่วนของผู้วิจัย)

บรรยากาศ บริบทในการสัมภาษณ์เป็นอย่างไร

....

....

....

อะไรที่เป็นประเด็นหลักที่ได้จากผู้ให้ข้อมูลรายนี้

....

....

....

ข้อค้นพบใหม่ที่ได้จากการสัมภาษณ์ในครั้งนี้ เมื่อเปรียบเทียบกับการสัมภาษณ์ในครั้งที่ผ่านมา

....

....

....

ข้อมูลที่โดดเด่น น่าสนใจ น่าประหลาดใจ หรือ ทำให้นักวิจัยมีความคิดที่ต่างออกไป

....

....

....

พบปัญหาอะไรในขณะสัมภาษณ์ เช่น ข้อคำถามที่ไม่ชัดเจน ข้อคำถามที่ลืมสอบถาม เป็นต้น

....

....

....

ประสบการณ์ที่ได้จากการสัมภาษณ์ในครั้งนี้

....

....

....

BUU-IRB Approved
14 Jun 2024
